# Changing Perceptions About the Dermatology Residency Application Process Among Applicants and Program Directors: 2020-2022

**DOI:** 10.7759/cureus.57864

**Published:** 2024-04-08

**Authors:** Fatuma-Ayaan B Rinderknecht, Kandice C Bailey, Danielle E Novack, Adena E Rosenblatt, Dana Dunleavy, Bobby D Naemi, Ilana S Rosman, Ammar Ahmed, Scott Worswick

**Affiliations:** 1 Dermatology, University of California San Francisco School of Medicine, San Francisco, USA; 2 Dermatology, Washington University School of Medicine, St. Louis, USA; 3 Dermatology, Icahn School of Medicine at Mount Sinai, New York, USA; 4 Dermatology, University of Chicago Medical Center, Chicago, USA; 5 Medical Education, Association of American Medical Colleges, Washington DC, USA; 6 Medical Education, Association of American Medical Colleges, Washington DC, USA; 7 Dermatology, Dell Medical School - The University of Texas, Austin, USA; 8 Dermatology, University of Southern California Keck School of Medicine, Los Angeles, USA

**Keywords:** covid19, diversity and equity in medicine, medical education, residency and internship, dermatology

## Abstract

Importance

Over the last two years, dermatology has undergone significant reforms in the residency application process in efforts to reduce applicant stress, increase equity, and due to the COVID-19 pandemic.

Objective

We aimed to determine applicant and program director (PD) perspectives in implementing these changes over the last two application cycles.

Design, setting, and participants

Anonymous online surveys were administered by the Association of American Medical Colleges (AAMC) to PDs and applicants from the 2021-2022 dermatology residency application cycle. These results were compared with similar online surveys distributed after the 2020-2021 cycle.

Results

Coordinated interview release was introduced in the 2020-2021 dermatology application cycle. At that time, 57% of PDs and 84% of applicants wished that more programs participated in the release, compared to 53% and 84%, respectively, in the 2021-2022 cycle. In 2021, 28% of PDs reported matching applicants from their home institution higher on their list compared to 14% in 2022. In 2021 and 2022, 94% of PDs reported that diversity was an explicit goal in their application process. However, in 2021, 33% of PDs reported that they matched no UIMs (underrepresented in medicine) in their cohort, which grew to 39% in 2022.

Conclusions

This study identifies key trends in applicant and PD perspectives associated with changes in the application process such as coordinated interview release, virtual interviews, and emphasis on diversity. Additional data is needed from subsequent cycles in order to determine the efficacy of these reforms.

## Introduction

Dermatology is one of the most competitive and least diverse specialties in medicine. Over the last two years, our specialty has implemented significant reforms in the residency application process in efforts to reduce applicant stress and costs, increase equity, and standardize elements of the cycle while accommodating for the COVID-19 pandemic [1]. Due to significant differences between the 2020-2021 and 2021-2022 application cycles, we aimed to compare them to understand the magnitude of implemented changes. In this study, we determined the applicant and program director (PD) perspectives on these changes over the last two application cycles. 

## Materials and methods

Two anonymous online surveys were sent out by the Association of American Medical Colleges (AAMC) using the Verint survey platform (Verint Systems, Melville, USA). The applicant survey was sent via email to 1046 dermatology applicants whose data was available on MyERAS (Electronic Residency Application Service (ERAS)) on April 1, 2022. The PD survey was sent out to 136 dermatology PDs on ERAS. The applicant survey was open for 3 weeks and the PD survey was open for 4 weeks. Both surveys took approximately 10 minutes to complete and collected feedback on demographics, match outcomes and opinions on the match process. Survey analysis utilizing descriptive and statistical methods was conducted using SPSS version 28 (IBM Corp, Armonk, USA). This study was approved by the American Institutes for Research Institutional Review Board (IRB)(FWA00001666).

The surveys from the 2020-2021 application cycle were collected and analyzed using Qualtrics (Qualtrics, Provo, USA). The applicant survey was sent out to applicants via the Dermatology Interest Group Association email listserv. The number of applicants who received the survey could not be calculated as the number of dermatology applicants on the listserv was unknown. The PD survey was sent out to 115 PDs on the Association of Professors of Dermatology listserv. The surveys were administered between March 29 through May 23, 2021. These surveys were similar in length and content to the surveys from the 2021-2022 cycle. The surveys were reviewed and approved by the University of Southern California (Los Angeles, California) IRB (approval #UP-21-00118).

The results of the 2020-2021 and 2021-2022 surveys were compared to assess trends in perception about the dermatology residency application process.

## Results

Applicant demographics 

The 2021-2022 and 2020-2021 applicant classes differed in terms of demographics. In 2020-2021, 85.3% of applicants were MD graduates, and 5.2% were DO graduates compared to 71% and 10%, respectively, in 2021-2022. In 2020-2021, 15.1% of applicants had a dual degree versus 8.1% in 2021-2022. In 2020-2021, 12.7% of applicants were Black, 8% Hispanic/Latino, and 48.5% White. In 2021-2022, 8.7% of applicants were Black, 6.8% Hispanic/Latino, and 59.7% White (Table 1).

**Table 1:** Applicant respondent characteristics and data

	2020-2021	2021-2022
Current status	(%)	(%)
US medical senior (MD)	85.3	71
US medical senior (DO)	5.2	10
International medical graduate	3	2
Current resident	3.4	4
Dermatology research fellow (post doctoral)	3	4
Dual Degree		
Yes	15.1	8.1
No	84.9	91.9
Sex		
Male	33.5	39.4
Female	65.2	60.6
Prefer not to answer	1.3	0
Race/ethnicity		
American Indian or Alaskan Native	0.8	0.6
Asian	20.7	22.3
Black, African American, or African	12.7	8.7
Hispanic, Latino, or of Spanish origin	8	6.8
White	48.5	59.7
Multiple races/ethnicities	2.5	n/a
Prefer not to answer	3.8	2.9
other	3	10.6
Home Dermatology Program	(%)	(%)
Yes	80.2	n/a
No	19.8	n/a
Re-applicant to dermatology		
Yes	8.7	9
No	91.3	88.4
Applications submitted		
<20	1.9	5.5
21-40	3.8	6.5
41-60	18.2	10.6
61-80	19.1	20.3
81-100	30.1	21.3
>100	26.8	35.8
Matched into dermatology		
Yes	82.8	77.2
No	17.2	22.8

Coordinated interview invite release

**Figure 1:**
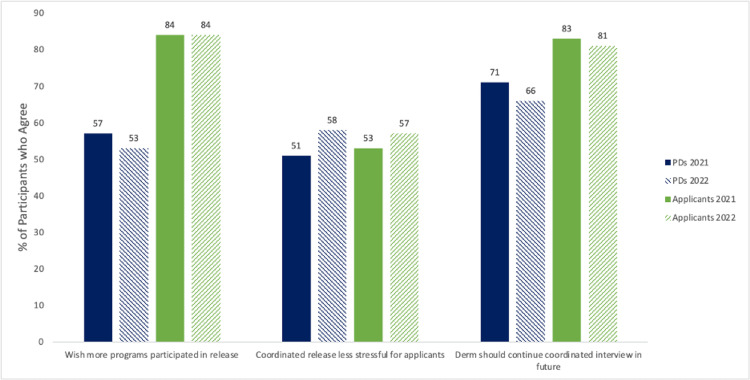
Impressions on coordinated interview invite release by dermatology PDs and applicants PD: program director

Coordinated interview invite release, in which residency programs release their interview invitations on the same days, was introduced in the 2020-2021 dermatology application cycle. PDs and applicants agreed across both cycles that the coordinated interview invite release process was clear and easy to understand and should be continued in the future (Figure 1). Although more programs participated in 2021-2022 compared to the prior cycle, applicants and PDs would like to see even greater participation among programs.

Future reforms

**Figure 2:**
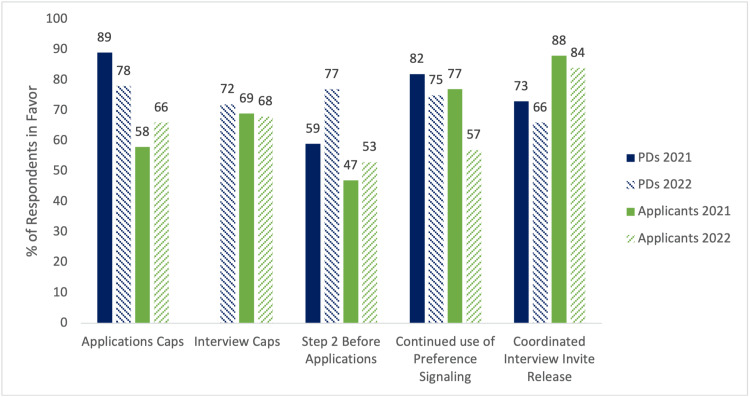
Support for future reforms in the dermatology match by dermatology PDs and applicants PD: program director

Applicants and PDs were surveyed on their support for possible future reforms in the application process (Figure 2). The top three reforms that PDs supported during the 2021-2022 cycle were application caps (setting a limit of how many applications per specialty can be submitted by each candidate), the requirement of Step 2 CK at the time of application (of note, Step 1 reporting moved to pass/fail in January 2022), and preference signaling (formal indication of a pre-specified number of preferred programs for each applicant at the time of application). The top three reforms supported by applicants during the 2021-2022 cycle were interview caps (setting a limit on how many interviews each applicant can complete in a specialty), application caps, and preference signaling. Overall, support remained high from both PDs and applicants for most of these proposed reforms.

Diversity in dermatology

In both cycles, 94% of PDs reported that diversity was an explicit goal in their application process. However, in the 2020-2021 cycle, one-third of PDs reported that they matched no UIM (underrepresented in medicine) applicants in their cohort; this grew to 39% in the 2021-2022 cycle (Table 2). Programs with 25-49% UIM applicants also fell in 2022 (21% vs 38% in 2020-2021), however, more programs reported matching 50-75% or all of their cohort from UIM groups. When considering non-racial diversity, applicants coming from low socioeconomic and first-generation backgrounds increased in 2022 compared to 2021 (Table 3).

**Table 2:** Program director reported match outcomes with respect to applicants underrepresented in medicine (UIM) status

Percentage of matched applicants were UIM	2020-2021 (%)	2021-2022 (%)
none	33	39
<25%	21	24
25-49%	38	21
50-75%	7	13
>75%	0	0
All	0	3

**Table 3:** Match results with respect to applicant background

	2020-2021 (%)	2021-2022 (%)
First in family to graduate college	7	14
First in family to attend professional/graduate school	22	36
Grew up in a rural area	12	20
First-generation immigrant	14	19
Low socioeconomic status (e.g., recipient of Medicaid, SNAP, Pell grants)	9	14

COVID-19 impact on dermatology match

In 2020, the majority of residency programs switched to virtual interviews and halted away rotations due to the COVID-19 pandemic. In the most recent application cycle (2021-2022), many programs reinstated away rotations but continued to hold virtual interviews. In the 2020-2021 cycle, 30% of PDs and 47% of applicants supported required in-person interviews after initial screening with virtual interviews; these numbers dropped to 16% and 33% in 2022.

When considering the effect of virtual interviews and limited away rotations on match data, 28% of PDs in 2020-2021 reported matching more applicants from their home institution than in a typical year, compared with 14% in 2022 (Table 4).

**Table 4:** Program director reported match outcomes with respect to applicants from their home institution

	2020-2021 (%)	2021-2022 (%)
Matched more students from our home institution than is typical	28	14
About the same	61	69
Matched fewer students from our home institution than is typical	10	17

## Discussion

While several changes have been made to the dermatology application process in the last two years, continued study of efficacy and support is important to determine best practices and future adjustments or new reforms.

Our study finds that coordinated interview invite release, now in its third year in the dermatology application process, continues to have strong support from both PDs and applicants. Other specialties that have incorporated coordinated interview invite releases have reported similar support [2,3] suggesting that this could become standard across specialties. In the most recent 2022-2023 application cycle, dermatology residency programs could choose to release interview invites on one of three pre-specified dates. As in the two prior cycles, there is a grace period that varies from 48 hours to 1 week after each invite release date to allow applicants time to consider their offers prior to accepting and scheduling. The number and frequency of coordinated interview invite release dates have been updated each cycle based on feedback from both program directors and applicants to maximize program participation and lower applicant anxiety throughout the process.

When considering other reforms that could be implemented in the dermatology residency application process to decrease costs to applicants and to optimize the alignment of programs and applicants, both PDs and applicants appear to support the integration of application caps and preference signaling. Dermatology residency candidates apply to an average of 77 programs per year [4], and program directors currently receive between 500-700 applications to screen each year [5]. Data shows that for US MD graduates with a mid-range Step 1 score (239-251), chances of matching into a dermatology program begin to plateau after applying to more than 42 programs [6]. However, given that Step 1 scores will now be reported as pass/fail, it will become more difficult for applicants to determine how many programs they should apply to. Because of the financial cost to applicants and the time cost to PDs, most of both groups support the introduction of application caps. Besides reducing costs to applicants, application caps could facilitate holistic review and theoretically would lead to better alignment between programs and applicants [7].

Preference signaling, which allows applicants to signal their interest to certain programs, was first implemented in dermatology during the 2021-2022 application cycle as part of the supplemental Electronic Residency Application Service (ERAS) application [8]. In the most recent cycle (2022-2023), a total of 16 specialties are participating in some form of preference signaling. While there is variability in the number and type of preference signals allotted in each specialty [9], applicants have an increased chance of receiving an interview invite at programs they have signaled across specialties [10,11], including in dermatology [12]. Taken together, the goal of application caps and preference signals is to better align applicants to their programs of interest while improving efficiency and equity in the process

In addition to reducing applicant stress in the application process, there has also been a push in dermatology to improve equity and diversity in the field [13]. Dermatology historically has been one of the least diverse specialties in the medical field [14]. In both the 2020-2021 and 2021-2022 cycles, 94% of PDs stated that diversity was an explicit goal in the match process. While the number of programs that did not match any UIM applicants increased in 2021-2022 compared to the prior year, there were also more programs that matched a higher proportion of their cohort from UIM groups (50-75% or 100% of the matched class). This suggests that there may be clustering of UIMs in a smaller number of programs. Whether this is due to program-specific recruitment practices or applicant preferences remains unclear. However, it is also important to note that in 2020-2021 there were higher percentages of Black and Hispanic/Latino applicants when compared to 2021-2022, and lower percentages of White applicants in 2020-2021 versus 2021-2022, which may provide some reasoning as to the reduction in matched UIM applicants in the latter cycle. Further, match outcomes data has not previously been racially stratified. Recently, the National Resident Matching Program (NRMP) began collecting demographic data during the match process [15] but has not yet released dermatology-specific information about match rates across racial and ethnic groups. Extrapolating our data to determine whether UIM applicants were more or less likely to match into dermatology is not possible.

Lastly, it is important to consider the effect that the COVID-19 pandemic continues to have on the dermatology application cycle and its potential effects on equity. In this study, we found that in 2020-2021, programs were more likely to match applicants from their home institution when compared to prior years and to the 2021-2022 cycle. Other studies also suggest that since the COVID-19 pandemic began, an increased number of applicants matched at their home institution [16]. Several factors could explain this phenomenon. There was a significant change in policies regarding in-person away rotations, with only rare rotations allowed in 2020-2021 and 1-2 per applicant allowed in 2021-2022. Given that these restrictions were - for the most part - lifted in 2022-2023, it will be interesting to compare “home” matching rates moving forward. Virtual interviews may also explain the increase in “home” matches, as there is less exposure to current residents and faculty and/or a decreased understanding of program culture through the virtual landscape. Lastly, given the uncertainty and anxiety associated with the pandemic, applicants may have preferred to stay in their current location where they presumably already have a support system and higher degree of stability. The changes in the “home” match rate are important to consider from an equity perspective, as previous data shows that UIM students are more likely to graduate from medical schools without home dermatology programs [17]. As the residency application landscape continues to change, it is important to consider how virtual interviews and away rotation policies impact geographic match trends and diversity in the field.

This study was limited by selection bias, as all PDs and applicants did not fill it out, and it is possible that those who did fill it out felt more strongly about particular issues. Additionally, it is difficult to make definitive statements on the match outcomes of UIM applicants without the National Resident Matching Program's racially stratified data. 

## Conclusions

This study identifies key trends in PD and applicant perspectives associated with reforms to the residency application process such as coordinated interview invite release, virtual interviews, and preference signals. Additional data is needed from subsequent cycles in order to determine the efficacy and popularity of these reforms. Ideally, correlating with outcomes data - such as interview and/or match data - would further enhance our understanding of best practices in the residency application process.
